# Association of *COL12A1* rs970547 Polymorphism with Elite Athlete Status

**DOI:** 10.3390/biomedicines10102495

**Published:** 2022-10-06

**Authors:** Valentina Ginevičienė, Alina Urnikytė

**Affiliations:** Department of Human and Medical Genetics, Biomedical Science Institute, Faculty of Medicine, Vilnius University, Universiteto Street 3, LT-01513 Vilnius, Lithuania

**Keywords:** athletic performance, *COL12A1*, rs970547, positive selection

## Abstract

The role of genetics, as an intrinsic factor, in research of sports performance increases with every passing year. The polymorphism rs970547 of the *COL12A1* gene is one of the most promising genetic markers linked to soft-tissue injuries. This study aimed to investigate whether *COL12A1* rs970547 genotypes are associated with elite Lithuanian athletes from high-risk various sports, such as running, throwing, jumping, and football. The study involved 293 Lithuanian elite athletes and 287 healthy untrained individuals from the Lithuanian population. The results of this study suggest that the rs970547 T allele and TT genotype were significantly over-represented in the total athlete group compared to controls (*p* < 0.05). There was a significantly lower C allele frequency in the sprint/power group (16.9%) as well as in footballers (19.4%) compared to controls (33.3%, *p* < 0.05). Positive selection analysis results showed that the derived allele experiences selection pressure within the general population of Lithuanians. Taken together, the findings of this study suggested that *COL12A1* rs970547 (T allele and TT genotype) is associated with elite athlete status, especially with sprint/power athlete and footballer`s performance. However, larger-scale studies within different ethnic backgrounds are still warranted to confirm the findings of our study.

## 1. Introduction

The benefits of human physical activity are well known. However, there is always a risk of exercise-related injuries. Musculoskeletal injuries occur frequently among athletes, fitness programme participants, and others who engage in routine vigorous exercise.

The etiology of exercise-related injuries is a complex and multifactorial phenomenon influenced by both intrinsic and extrinsic factors: environmental, genetic, anatomical, physiological, and psychological. Currently, researchers are looking for ways to improve the performance of athletes and reduce or prevent the risk of injury [1].

In recent years, scientists are increasingly paying attention to the influence of genetic factors on competitive success, physical performance traits, and risk of injuries [1,2,3,4]. In the field of exercise genetics, research studies identified over 200 genetic markers potentially linked with some physical performance phenotypes [1]. Differences in structure, function, and regeneration between muscle, ligament, and tendon suggest there are likely to be tissue-specific genetic markers, some of which may affect physical performance (e.g., flexibility) and injury risk, or the recovery once the injury has occurred. Studies investigating genetic markers for sports injury risk have identified more than 80 loci associated with a range of complaints. Moreover, 29 genetic markers have been independently associated with soft-tissue injury in two or more studies, of which *COL1A1* rs1800012, *COL5A1* rs12722, *COL12A1* rs970547, *MMP1* rs1799750, *MMP3* rs679620, and *TIMP2* rs4789932 are most promising [1]. The majority of replicated markers were identified using hypothesis-driven studies targeting genes encoding proteins involved in biological and molecular processes. These include specific single nucleotide polymorphisms (SNP) of genes influencing the soft-tissue structure and affecting the mechanical properties of the muscle-tendon unit, e.g., genes encoding the structural, collagen type I, V, and XII proteins (COL1A1, COL5A1, COL12A1) [2,5,6]. Collagens are a large family of structural proteins composed of 28 types of collagen, the main components of structural connective (tendons, ligaments, and other components of musculoskeletal tissues), and epithelial tissues, accounting for about 20% of total body weight [7]. The collagen family can be divided into two groups, based on their structure and function: (1) the fibrillar collagens which form the fibrillar scaffolding for the extra-cellular matrix and, (2) the non-fibrillar collagens which include, the fibril associated collagens with interrupted triple helices (FACITs), network forming collagens, short chain collagens, and beaded filament collagens. Studies have shown that sex hormones play a role in the regulation of collagen synthesis and degradation in human ligament tissue [7,8].

Types XII collagen belongs to a family of non-fibrillar collagens that are also associated with the surface of the collagen fibril and are members of the FACITs subfamily. The COL12A1 gene, mapped to chromosome 6q12-q13, encodes the alpha1 chains of the long (XIIA) and short (XIIB) homotrimeric isoforms of collagen type XII [2,5,8]. The main functions of type XII collagen are: participates in the formation of fibers together with type XV collagen; forms internal interfibrillar connections and regulates the interaction of collagen fibers with intracellular and the cell surface in ligaments; regulates the response to mechanical pressure; during wound healing, this type of collagen increases along with collagen types V and III [8]. According to the database of the National Center for Biotechnology and Information (NCBI), five SNPs of COL12A1 exonic regions were identified of which only two (rs240736 and rs970547) are considered to be non-synonymous [5]. The polymorphism rs970547 (NM_080645.2:c.5680T>C, NP_004361.3:p.Gly3058Ser) in exonic regions (terminal 65th exon) of COL12A1 is one of the most promising genetic markers linked to soft-tissue injuries (generally—anterior cruciate ligament rupture) [1], mainly described in South African and Polish populations [6,9]. It has been reported that the rare C allele and CC genotype of *COL12A1* rs970547 is significantly overexpressed in a patient with joint laxity and anterior cruciate ligament (ACL) ruptures, but other reports suggest that there is no association between ACL ruptures [9,10,11]. The current level of evidence is not strong enough to confirm rs970547 polymorphism for injury risk. More research is needed to understand this genetic variant contributing to sports performance and injury risk. Moreover, exercise-induced injuries to the musculoskeletal system depending on race, age, body composition, physical activity/inactivity levels, and their multifactorial interaction with other extrinsic and intrinsic factors [12].

Given the high incidence of injuries in elite athletes, we hypothesised that COL12A1 rs970547 polymorphism would be associated with career success. The present study aimed to investigate the association between the COL12A1 rs970547 genotypes and elite Lithuanian athletes from high-risk various sports to answer the question of whether or not the COL12A1 rs970547 polymorphism is important concerning elite athletes’ status in our population. We also checked if positive natural selection has been acting on this polymorphism.

## 2. Materials and Methods

### 2.1. Samples

The study involved 293 Lithuanian elite athletes (247 males and 46 females, aged 31.5 (±6.03)) and 287 controls (190 males and 97 females, aged 32.2 (±4.25), healthy, unrelated Lithuanian individuals who were not athletes). The athletes were classified into three groups (as determined by the distance, duration, and energy requirements of their sport): (1) endurance-oriented athletes competing in long distance, and duration events demanding predominantly aerobic energy production—rowers, 5–10 km, and marathon runners (*n* = 66), (2) sprint/power-oriented athletes, whose events demand predominantly anaerobic energy production—track and field sprinters (100–400 m running, throwing and jumping, *n* = 80); (3) team-sport which requires both the anaerobic and aerobic energy production (footballers, *n* = 147). All athletes were ranked in the top 10 nationally in their sports discipline: elite-level (participated in major international competitions, including the European Championships, World Championships, and Olympic Games), and only footballers ranked in ‘national-level’ participated in national competitions. Inclusion criteria stipulated that athletes must have no history of positive tests for performance-enhancing substances under standard anti-doping controls. All participants were Caucasians.

Following the Declaration of Helsinki, informed consent was obtained from all the study participants. All procedures in this study conformed with ethical standards concerning scientific research of sport and exercise and were approved by the Lithuanian Bioethics Committee.

DNA was obtained from whole blood using either a standard phenol-chloroform method of extraction or the automated DNA extraction platform TECAN Freedom EVO (TECAN Group Ltd., Eppendorf, Switzerland). A NanoDropR ND-1000 spectrophotometer (NanoDrop Technologies Inc., Wilmington, DE, USA) was used to assess DNA concentration and quality.

### 2.2. Genotyping of COL12A1 Gene SNP rs970547

Genotyping for *COL12A1* rs970547 polymorphism was performed in duplicate using an allelic discrimination assay on a 7900HT Fast real-time polymerase chain reaction instrument (Applied biosystems™, Life Technologies, 2012, Carlsbad, CA, USA) with TaqMan probes (TaqMan^®^ Pre-Designed SNP Genotyping Assay ID: C_7580617_10). Genotypes were assigned using software (SDS Software v2.3, Applied Biosystems^TM^). To ensure proper internal control, positive and negative controls from different DNA aliquots were used for each genotype analysis. The average genotyping rate was ~98%.

### 2.3. Statistical Analysis

Genotype frequencies of athletes and non-athletes controls were tested for compatibility with Hardy-Weinberg equilibrium (HWE). Differences in genotype and allele frequency were compared using the chi-square goodness-of-fit test. The homogeneity hypothesis for genotype and allele frequency differences between groups were assessed by Pearson’s chi-squared exact test. Odds ratios (OR) with 95% confidence intervals (CIs) were calculated where there was a significant difference in genotype distribution between groups. The level of significance was set at *p* < 0.05. All analysis was performed using the R Studio v.3.4 and SPSS statistical software package (IBM SPSS v.21).

### 2.4. Selection Analysis

To verify whether the COL12A1 gene variant rs970547 is affected by microevolutionary factors in the Lithuanian population, we performed a positive selection analysis using whole genome sequencing data of 50 Lithuanian individuals downloaded from https://figshare.com/articles/dataset/Inherited_and_de_novo_variation_in_Lithuanian_genomes_introduction_to_the_analysis_of_the_generational_shift 19354817 (accessed on 14 March 2022) (Urnikyte et al. 2022).

Signatures of recent selection were estimated using the cross-population extended haplotype-based homozygosity (XP-EHH) test [13] that was computed between Lithuanians and reference populations (CEU, FIN, YRI) obtained from the 1000 Genomes Project Phase3 dataset [14]. After merging we left with 1,443,372 common SNPs. Older signals of selection were detected with Tajima’s D statistic [15]. For the construction of haplotypes, SHAPEIT2 software was used. XP-EHH was run using Selscan v1.2.0a [16], and Tajima’s D with the PopGenome package [17] was implemented in R considering 100 kb sliding windows with a step size of 10 kb. We kept SNPs with XP-EHH >2 as indicative of selection. Negative Tajima’s D values were identified considering the rank of the score in the genomic distribution. Windows were sorted in ascending order and we considered for further analysis those with empirical *p*-values less than 0.01. The regions under selection were annotated with ANNOVAR [18] using GRCh37 (hg19), dbSNP147 [19], RefSeqGene, and CADD (Combined Annotation Dependent Depletion) version 1.347 [20].

## 3. Results

### 3.1. Case-Control Study

The distribution of *COL12A1* rs970547 polymorphism allele and genotype frequencies in 293 elite athletes was compared to 287 healthy untrained individuals from the Lithuanian population. Data of genotype and allele distribution analysis is presented in Table 1. Results showed that the genotype distributions were in line with HWE for athletes (*p* > 0.05), however, the deviation was observed in controls (*p* = 0.00005).

All analyses included an adjustment for sex. The *COL12A1* rs970547 frequencies of genotypes revealed significant differences between the non-athlete Lithuanian male and female groups (TT/TC/CC: 54.2/33.2/12.6% vs. 41.2/35.1/23.7%, *p* = 0.047). The rs970547 minor (injury risk allele) C allele was significantly over-represented in the female (41.2%) group compared to the male (29.2%, *p* = 0.005) from the general population of Lithuania. These findings suggest that rs970547 polymorphism is gender-specific in the Lithuanian population.

The results of the case-control association analyse showed that rs970547 allele and genotype frequencies were significantly different between the total athlete group and the controls (*p* < 0.05). The rs970547 C allele (33.3%) and CC genotype (16.4%) appeared to be more frequent in controls in comparison to the athletes (C allele 20%; CC genotype 5.8%; *p* < 0.05). Furthermore, sprint/power-oriented athletes and footballers had significantly different allele and genotype frequencies compared to controls (*p* < 0.05). There was a significantly lower C allele frequency in the sprint/power group (16.9%) as well as in footballers (19.4%) compared to controls (33.3%, *p* < 0.05). Sprint/power athletes and footballers were more likely than controls to possess the T allele, with a greater likelihood of having the TT genotype compared to the TC + CC (sprint/power [OR = 0.45; CI [0.27–0.76]; *p* = 0.0024); footballers (OR = 0.45; CI [0.30–0.69]; *p* = 2 × 10^−04^]), and TT + TC genotypes compared to the CC in sprint/power group [OR = 0.13; CI = [0.03–0.55]; *p* = 2 × 10^−04^] and in footballers [OR = 0.41; CI = [0.21–0.82]; *p* = 0.0072]). These results indicate that the T allele may be beneficial for sprint/power athletes and footballers compared to non-athletes.

According to the gender analysis (in athletes group), for male subjects, the rs970547 frequencies of allele and genotypes revealed significant differences between the total athletes and non-athlete male groups (T/C alleles: 79.9/20.1%; TT/TC/CC: 65.2/29.5/5.4% vs. T/C alleles: 70.8/29.2%, *p* = 0.002; TT/TC/CC: 54.2/33.2/12.6%, *p* = 0.004). Moreover, for female subjects, there were significant difference on allele and genotype distributions between total athletes and non-athletes female groups (T/C alleles: 80.4/19.6%; TT/TC/CC: 67.4/26.1/6.5% vs. T/C alleles: 58.8/41.2%, *p* = 0.0005; TT/TC/CC: 41.2/35.1/23.7%, *p* = 0.001). Thus, rs970547 T allele and TT genotype were significantly over-represented in athletes compared to controls (for both groups—male and female). Furthermore, the T allele was significantly higher in male sprint/power-oriented athletes (83.3%) and footballers (80.6%) compared to male controls (70.8%, *p* < 0.05).

### 3.2. COL12A1 rs970547 Polymorphism Adaptation in the Lithuanian Population

By analysing recent signatures of population positive selection (whole genome sequencing data of 50 non-athletes Lithuanian individuals), in the LT-YRI (Lithuania-Yoruba) comparison, we detected a 145 kb region in chromosome 6, comprising the *COL12A1* gene. Interestingly, a non-synonymous variant rs970547 (NC_000006.11:g.75797302C>T), with a CADD value (Combined Annotation Dependent Depletion—tool scores the predicted deleteriousness of SNPs in the human genome by integrating multiple annotations including conservation and functional information) 27.8 was found among the significant XP-EHH values. XP-EHH test is powerful for detecting selection signatures at or near fixation by comparing haplotypes from two populations. Results indicate that European and African populations present high frequencies for the derived T allele at the analysed rs970547 variant, 0.77 and 0.67 respectively. The region containing the COL12A1 gene for older signatures of selection was also identified, with a *p*-value of 0.006.

## 4. Discussion

The role of genetics, as an intrinsic factor, in research of sports performance increases with every passing year. In our study, we investigated the association between the *COL12A1* rs970547 genotypes and elite Lithuanian athletes from high-risk various sports, such as various distances running, throwing, jumping, and football. One of the main findings was that rs970547 deviated from HWE in the group of not athletes’ Lithuanian individuals (controls). That might have been due to an excess of the rare C allele in the control group, especially in females (41.2%, compared to males 29.2%). This deviation seems responsible for differences in genotypic distributions in the Lithuanian population for the rs970547 polymorphism. Findings suggest that rs970547 polymorphism is gender-specific. Disturbances of the HWE occur when microevolutionary factors, such as natural positive selection favors a particular genotype giving a differential and specific trait to an individual. More often, HWE indicates population stratification. One of the possible causes of HWE departure in our results is genetic stratification, all the non-athlete individuals included in this study were strictly selected (healthy, unrelated Lithuanian individuals from six ethnolinguistic regions). We also checked if a positive selection has been acting on this polymorphism. The results showed that the derived T allele experiences selection pressure within the general population of Lithuanians. This suggests that the T allele of the variant rs970547 may confer a selective advantage, which can be linked to enhanced athletic performance. Additional investigation is required to test this hypothesis.

In the athlete group, the HWE was preserved. The main findings of this study were that rs970547 T allele and TT genotype were significantly over-represented in the total athlete group compared to controls (for both groups—male and female) (*p* < 0.05). Furthermore, sprint/power athletes and footballers were more likely than controls to possess the T allele and TT genotype (*p* < 0.05). The rs970547 rare C allele and CC genotype appeared to be more frequent in controls in comparison to the athletes (*p* < 0.05).

Because *COL12A1* rs970547 polymorphism is a non-synonymous coding variant (causing amino acid at position 3058 to change from serine to glycine), some investigators suppose that the amino acid replacement may alter the biomechanical properties of the collagen fibril and affect the occurrence of musculoskeletal soft tissues (muscles, tendons, and ligaments) injury. However, till now, no conclusive evidence could be found to suggest the functional consequence of this change in amino acid sequence [21]. In the past years, several case-control genetic association studies have been performed to investigate the associations between rs970547 polymorphism and ACL injury risk among different populations (African, Asian and Caucasian), but studies did not yield consistent results [21]. Posthumus and colleagues (2010) for the first time investigated whether the rs970547 polymorphism was associated with ACL rupture in South African participants and found that the rs970547 polymorphism (TT genotype) was significantly associated with the occurrence of ACL rupture in females (from non-contact jumping sports) and not in male (from contact sports) [22]. Ficek et al., [4] and O’Connell et al., [9] concluded similar results, that the TT genotype of rs970547 was associated with increased risk in female, but not male participants or the whole population (the main subjects were Caucasians). Moreover, the female-specific genetic associations observed between joint laxity and rs970547 are consistent with the female-specific genetic associations with ACL injury for rs970547. These associations may reflect a potential hormone-gene interaction, given the known effects of sex hormones on collagen metabolism and the potential for estrogen to interact with mechanical tension to deferentially regulate gene expression and, thus, the abundance of collagen [23]. For the remaining studies, the conclusions were controversial to the above studies. The results from John et al. [11] and Kang et al. [24] showed that both the T allele and the TT genotype frequency were at significantly higher risk of ACL rupture in the whole population. In a recent study, the authors identified that male individuals carrying the *COL12A1* rs970547 T allele and TT genotype may have an increased risk of ACL rupture [25]. In meta-analysis [21], the data confirmed that the rs970547 polymorphism was not related to the risk of ACL injuries, and in stratification analyses by ethnicity or sex, this conclusion was confirmed once again. Furthermore, in the results of a previous study to determine the effect of rs970547 in Achilles tendon injury, no statistically significant differences were identified in the genotype or allele distributions between the affected and control subjects [26].

Our study was conducted in a Caucasian population (Lithuanian elite athletes and non-athletes individuals). More high-quality studies are needed to verify the genetic impact of *COL12A1* polymorphism in elite sports performance and the risk of injuries that occur during physical activities. If the association and effect of rs970547 in athletic performance could be confirmed by future studies (within different ethnic backgrounds), it would be of great interest to dive deeper into the possible link between *COL12A1* polymorphisms and pathogenesis of musculoskeletal soft-tissue injuries.

There are several limitations to this study. Because of the hypothesis-driven nature of this study, the sample size is relatively small. Participants’ nationalities were restricted to Lithuanian, meaning the associations described in the present study cannot be generalized to athletes from other countries or geographical ancestries. We also did not collect phenotype data (physiological measures, information about injuries, etc.) of athletes. However, our inclusion criteria stipulated that all athletes had competed at the national or international level, which would not be possible without the physiological characteristics associated with elite athlete phenotypes. Furthermore, only one SNP was studied, and given the number of genes that have the potential to influence athletic performance, additional relevant genetic variants should be explored. We need to extend these findings in athlete groups by performing WGS (Whole genome sequencing) or genome-wide genotyping analysis.

Taken together, the findings of this study suggested that *COL12A1* rs970547 T allele and TT genotype is associated with elite athlete status, especially with sprint/power athletes and footballer performance. However, larger-scale studies within different ethnic backgrounds are still warranted to confirm the findings of our study.

## 5. Conclusions

The present study reports the association of the *COL12A1* rs970547 with elite status in Lithuanian athletes from high-risk various sports, especially sprint/power athletes and footballers. Therefore, the *COL12A1* rs970547 polymorphism may be considered a candidate gene to influence the performance of elite athletes.

## Figures and Tables

**Table 1:** Genotype and allele frequencies of the COL12A1 rs970547 polymorphism in athletes and non-athletes from Lithuania.

Sample		*n*	Allele Frequency (%)		*p*-Value vs. Non-Athletes	Genotype Frequency (%)			*p*-Value vs. Non-Athletes
			T	C		TT	TC	CC	
Sprint/power	Total	80	83.1	16.9	0.00009 *	68.8	28.8	2.5	0.0003 *
	Female	20	82.5	17.5	0.00807 *	70.0	25.0	5.0	0.0126
	Male	60	83.3	16.7	0.00918 *	68.3	30.0	1.7	0.0115
Endurance	Total	66	75.0	25.0	0.08200	56.1	37.9	6.1	0.093
	Female	26	78.9	21.2	0.01230	65.4	26.9	7.7	0.0188
	Male	40	72.5	27.5	0.86410	50.0	45.0	5.0	0.7742
Footballers	Total	147	80.6	19.4	0.00003 *	68.7	23.8	7.5	0.00012 *
	Female	NA	NA	NA	NA	NA	NA	NA	NA
	Male	147	80.6	19.4	0.00003 *	68.7	23.8	7.5	0.00012 *
All athlete	Total	293	80.0	20.0	4.07 × 10^−7^ *	65.9	28.3	5.8	2.91 × 10^−6^ *
	Female	46	80.4	19.6	0.00051 *	67.4	26.1	6.5	0.0014 *
	Male	247	79.9	20.1	0.00217 *	65.6	28.7	5.7	0.00349 *
All non-athlete	Total	287	66.7	33.3	NA	49.8	33.8	16.4	NA
	Female	97	58.8	41.2	NA	41.2	35.1	23.7	NA
	Male	190	70.8	29.2	NA	54.2	33.2	12.6	NA

* Significant differences between the athlete groups and control group (non-athletes).

## Data Availability

The data presented in this study are available on request from the corresponding authors.

## References

[1] Ahmetov I.I., Hall E.C.R., Semenova E.A., Pranckevičienė E., Ginevičienė V. (2022). Advances in Sports Genomics. Advances in Clinical Chemistry.

[2] Dines H.R., Nixon J., Lockey S.J., Herbert A.J., Kipps C., Pedlar C.R., Day S.H., Heffernan S.M., Antrobus M.R., Brazier J. (2022). Collagen Gene Polymorphisms Previously Associated with Resistance to Soft-Tissue Injury Are More Common in Competitive Runners Than Nonathletes. J. Strength Cond. Res..

[3] Perini J.A., Lopes L.R., Guimarães J.A.M., Goes R.A., Pereira L.F.A., Pereira C.G., Mandarino M., Villardi A.M., de Sousa E.B., Cossich V.R.A. (2022). Influence of Type I Collagen Polymorphisms and Risk of Anterior Cruciate Ligament Rupture in Athletes: A Case-Control Study. BMC Musculoskelet. Disord..

[4] Ficek K., Stepien-Slodkowska M., Kaczmarczyk M., Maciejewska-Karlowska A., Sawczuk M., Cholewinski J., Leonska-Duniec A., Zarebska A., Cieszczyk P., Zmijewski P. (2014). Does the A9285g Polymorphism in Collagen Type XII A1 Gene Associate with the Risk of Anterior Cruciate Ligament Ruptures?. Balk. J. Med. Genet..

[5] Dalewski B., Kaczmarek K., Jakubowska A., Szczuchniak K., Pałka Ł., Sobolewska E. (2021). COL12A1 Single Nucleotide Polymorphisms Rs240736 and Rs970547 Are Not Associated with Temporomandibular Joint Disc Displacement without Reduction. Genes.

[6] Posthumus M., September A.V., Keegan M., O’Cuinneagain D., Van der Merwe W., Schwellnus M.P., Collins M. (2009). Genetic Risk Factors for Anterior Cruciate Ligament Ruptures: COL1A1 Gene Variant. Br. J. Sports Med..

[7] Sherman V.R., Yang W., Meyers M.A. (2015). The Materials Science of Collagen. J. Mech. Behav. Biomed. Mater..

[8] Keene D.R., Lunstrum G.P., Morris N.P., Stoddard D.W., Burgeson R.E. (1991). Two Type XII-like Collagens Localize to the Surface of Banded Collagen Fibrils. J. Cell Biol..

[9] O’Connell K., Knight H., Ficek K., Leonska-Duniec A., Maciejewska-Karlowska A., Sawczuk M., Stepien-Slodkowska M., O’Cuinneagain D., van der Merwe W., Posthumus M. (2015). Interactions between Collagen Gene Variants and Risk of Anterior Cruciate Ligament Rupture. Eur. J. Sport Sci..

[10] O’connell K., Posthumus M., Collins M. (2013). No Association between *COL3A1*, *COL6A1* or *COL12A1* Gene Variants and Range of Motion. J. Sports Sci..

[11] John R., Prabhakar S., Singh Dhillon M., Anand A., Minhas G. (2019). Association of ACL Tears and Single Nucleotide Polymorphisms in the Collagen 12 A1 Gene in the Indian Population—A Preliminary Case-Control Study. Muscle Ligaments Tendons J..

[12] Ginevičienė V., Utkus A., Pranckevičienė E., Semenova E.A., Hall E.C.R., Ahmetov I.I. (2022). Perspectives in Sports Genomics. Biomedicines.

[13] Sabeti P.C., Varilly P., Fry B., Lohmueller J., Hostetter E., Cotsapas C., Xie X., Byrne E.H., McCarroll S.A., Gaudet R. (2007). Genome-Wide Detection and Characterization of Positive Selection in Human Populations. Nature.

[14] Delaneau O., Zagury J.-F., Pompanon F., Bonin A. (2012). Haplotype Inference. Data Production and Analysis in Population Genomics.

[15] Tajima F. (1989). DNA Polymorphism in a Subdivided Population: The Expected Number of Segregating Sites in the Two-Subpopulation Model. Genetics.

[16] Szpiech Z.A., Hernandez R.D. (2014). Selscan: An Efficient Multithreaded Program to Perform EHH-Based Scans for Positive Selection. Mol. Biol. Evol..

[17] Pfeifer B., Wittelsbürger U., Ramos-Onsins S.E., Lercher M.J. (2014). PopGenome: An Efficient Swiss Army Knife for Population Genomic Analyses in R. Mol Biol Evol.

[18] Wang K., Li M., Hakonarson H. (2010). ANNOVAR: Functional Annotation of Genetic Variants from High-Throughput Sequencing Data. Nucleic Acids Res..

[19] Pruitt K.D., Tatusova T., Maglott D.R. (2007). NCBI Reference Sequences (RefSeq): A Curated Non-Redundant Sequence Database of Genomes, Transcripts and Proteins. Nucleic Acids Res..

[20] Kircher M., Witten D.M., Jain P., O’Roak B.J., Cooper G.M., Shendure J. (2014). A General Framework for Estimating the Relative Pathogenicity of Human Genetic Variants. Nat. Genet..

[21] Lv Z., Wang W., Zhao D., Huang J. (2021). COL12A1 Rs970547 Polymorphism Does Not Alter Susceptibility to Anterior Cruciate Ligament Rupture: A Meta-Analysis. Front. Genet..

[22] Posthumus M., September A.V., O’Cuinneagain D., van der Merwe W., Schwellnus M.P., Collins M. (2010). The Association between the COL12A1 Gene and Anterior Cruciate Ligament Ruptures. Br. J. Sports Med..

[23] Bell R.D., Shultz S.J., Wideman L., Henrich V.C. (2012). Collagen Gene Variants Previously Associated with Anterior Cruciate Ligament Injury Risk Are Also Associated with Joint Laxity. Sports Health Multidiscip. Approach.

[24] Kang B.Y., Eun S.P., Kim H., Lee J.K. (2020). Distributions of Genetic Polymorphisms in COL12A1 and MMP12 Genes in Korean Patients with Anterior Cruciate Ligament Rupture by Recreational Sports Activities. Med. Sport.

[25] Zhao D., Zhang Q., Lu Q., Hong C., Luo T., Duan Q., Shu S., Lv J., Zhao W. (2020). Correlations Between the Genetic Variations in the COL1A1, COL5A1, COL12A1, and β-Fibrinogen Genes and Anterior Cruciate Ligament Injury in Chinese Patientsa. J. Athl. Train..

[26] September A., Posthumus M., van der Merwe L., Schwellnus M., Noakes T., Collins M. (2008). The *COL12A1* and *COL14A1* Genes and Achilles Tendon Injuries. Int. J. Sports Med..

